# One Thousand Consecutive Inductions of General Anæsthesia

**Published:** 1900-09

**Authors:** C. Hamilton Whiteford

**Affiliations:** Anæsthetist to the South Devon and East Cornwall Hospital


### ONE THOUSAND CONSECUTIVE INDUCTIONS OF GENERAL ANÆSTHESIA.

BY

C. HAMILTON WHITEFORD, M.R.C.S., L.R.C.P.,
*Anæsthetist to the South Devon and East Cornwall Hospital.*

---

THE following remarks are based on a consecutive series which, excluding anæsthesia with nitrous oxide, is composed as follows :—

| | |
|---|---|
| Chloroform ... ... ... ... | 792 |
| Ether ... ... ... ... ... | 10 |
| A.C.E. ... ... ... ... ... | 4 |
| Nitrous oxide and ether ... ... | 17 |
| Chloroform and ether ... ... ... | 101 |
| Chloroform, A.C.E. and ether ... ... | 6 |
| A.C.E. and ether ... ... ... | 5 |
| A.C.E. and chloroform ... ... ... | 53 |
| Nitrous oxide, ether and chloroform ... | 1 |
| Methylene ... ... ... ... | 11 |
| | 1000 |

### CHLOROFORM.

I usually administer chloroform from lint spread on a wire frame, practically Skinner's inhaler, and sometimes give it from a Clover's inhaler without the bag. The most unpromising cases for an anæsthetic often take chloroform exceedingly well. Some years ago I gave chloroform, with some inward misgivings as to the result, to a man with a fracture in the lower cervical region. The surgeon kept him turned over nearly flat on his abdomen and performed laminectomy. He only had his diaphragm with which to respire, but his condition never gave a moment's anxiety, as he breathed perfectly throughout the operation.

In patients cyanosed from lung affections the combination of oxygen with chloroform is often of service, as in the following case.

The patient, a boy aged 15, had an abscess of the right lung, caused by a foreign body, which was discharging through a bronchus into the trachea. Chloroform was given for resection of ribs and drainage of abscess. At frequent intervals he coughed up fœtid pus and became black in the face. The cyanosis was treated by giving oxygen throughout the operation, which lasted three-quarters of an hour, the tube from the cylinder being inserted into one nostril, only enough chloroform being given to maintain a partial anæsthesia. In the case of deep cyanosis at the end of an operation, when the anæsthetic has been stopped, a less wasteful method is to attach the tube from the oxygen cylinder to a Rumboll-Birch ether inhaler.

A very disagreeable complication when chloroform is given at night near a lighted gas-jet is the decomposition of the vapour into carbonyl chloride, which, in the presence of moisture, is split up into carbonic and hydrochloric acids. This has happened to me on three occasions, and produced violent coughing in everyone present. In one case the pungent odour was so strong that the operator, assistant, and nurse had to leave the room. A nurse is reported to have died from inhaling these fumes while assisting at an operation.

In those cases where the patient, being only half anæsthetised, passes into a state of partial apnœa, the colour and pulse remaining good, I have found a minute's artificial respiration restore regular respiration with corresponding absorption of the anæsthetic and saving of time.

### ETHER.

The smell and taste of ether is so objectionable to many patients that I never now give it unless preceded by nitrous oxide or chloroform. I have seen no case of what is known as "ether pneumonia," but, where possible, avoid ether in patients with marked bronchitis. When using the Rumboll-Birch ether inhaler the patient's breath sometimes condenses freely in the glass chamber, where it can be seen as a distinct layer, on which the ether floats. On one occasion I was surprised to find the patient rapidly regaining consciousness in the middle of an operation with half an ounce of ether left in the glass chamber. On closer inspection the fluid was seen to be practically water, all the ether having evaporated.

When during chloroform anæsthesia the breathing or pulse has become bad, I have found nothing so satisfactory as ether given from a Clover's inhaler. Its one drawback is that the operator, especially in cases of intestinal obstruction, is sometimes tempted by the temporary improvement caused by the ether to perform a longer operation than is justified by the patient's real condition. It must never be forgotten that in these cases the patient is going at top pressure, and when a few hours later the stimulus of the ether wears off the resulting collapse is very great and sometimes fatal. In desperate emergency cases, ether, from the anæsthetist's point of view, is invaluable; it has often saved me from having a death on the table, although the patient has not infrequently succumbed within the next twenty-four hours. Young infants take ether well: I usually administer it to them from a mask, after producing partial anæsthesia with chloroform or A.C.E.

### IMMUNITY TO ANÆSTHETICS.

I have not seen a case in which anæsthesia could not be produced. Two cases of partial, probably acquired, immunity have come under my notice. The first is a personal experience. Eighteen months ago, at a time when I had been administering weekly a large number of anæsthetics and no doubt inhaling a certain amount myself, I had nitrous oxide and ether administered to me from a Rumboll-Birch inhaler. I lost consciousness with the gas, but as soon as the ether was mixed with the gas began to come round, and after a few minutes, during which I inhaled half an ounce of ether, was wide awake. Chloroform was then given, and I was easily anæsthetised. It seems probable that I had established a partial immunity by inhaling small doses for many days in succession. The second case is that of a medical friend, who, after being anæsthetist to a hospital for a year, required an enormous amount of chloroform, the administration of which lasted 1¼ hours to produce anæsthesia.

### TAIT'S MIXTURE.

Tait's mixture, two parts of ether to one of chloroform, given from a Clover's inhaler without the bag, I find to produce very satisfactory anæthesia in women.

### DEATH.

I have had one death during anæsthesia. The patient, a young woman, had a large pyo-nephrosis removed under chloroform. At the end of the operation the anæsthetic was stopped, and the dressings were being applied when the patient suddenly became rigid; pulse and respiration, which up to this point had been very fair, ceased *instanter*. The rigidity resembled the rigid stage of an epileptic attack. There was no history of epilepsy, and nothing abnormal was found at the *post mortem*. I have often speculated whether a large dose of toxins could have been absorbed from the suppurating kidney during the operation and produced the fit. But at best this is only conjecture. A fit is a rare mode of death during anæsthesia.

### TREATMENT OF EMERGENCIES.

Judging by some of the cases reported from time to time in medical literature, medical men occasionally lose their heads on the occurrence of an emergency during anæsthesia, and in some cases the loss of reasoning power persists to such an extent that they publish the case, with the treatment adopted, as being worthy of imitation. Only mental aberration will account for cases reported within the last few years where a patient is given ether, becomes cyanosed with feeble pulse, is then hung head downwards, and has more ether injected under the skin; or where, for a patient known to have œdema of the glottis, ether, which effectually closes the already narrowed rima, is selected as the anæsthetic; entry of air is mechanically prevented, yet artificial respiration is attempted for a quarter of an hour, but no effort is made to open the trachea. Such cases as the above are the result of unreasoning and indiscriminate application of routine methods.

The ordinary treatment of emergencies arising during anæsthesia is too well known to be entered into here, but there are two causes of collapse and their treatment to which I will refer :—1. Cooling of the patient by exposure of either the cutaneous surface or the viscera. 2. Loss of blood.

To prevent the first, care should be taken to see that the patient is thoroughly warm before the anæsthetic is given. It is illogical to anæsthetise a patient in a cold room on a cold table and then carry him to a warm operation table in the hope of warming him again, as the exposure necessary for the operation effectually neutralises any additional warmth which may be obtained from the table. When the operation table is warmed by such means as a hot-water bed, it is essential during a long operation to test from time to time the heat of the water, and when it cools to have it partly replaced by hotter water or supplemented by extra hot bottles. The water bed must always be hotter than the patient; it will not get absolutely cold, as it is being kept warm by the patient, who loses a corresponding amount of heat in the process. Stimulants, such as brandy and strychnine, injected hypodermically, by improving the circulation help to maintain the warmth of the patient. Hypodermic injections for collapse should be made deeply into muscles of the trunk, or of a limb near the trunk, and not beneath the skin of the extremities where, in a collapsed patient, the lymph circulation is practically *nil*. The pectoralis major is a useful muscle into which to inject, especially if artificial respiration is being performed, as the movements largely help to massage the fluid on into the general circulation. It is illogical to inject ether into a patient who has been inhaling ether ; he is already saturated.

To remedy both the first and second, intravenous injections of hot sterile saline solution, up to six or more pints, with the addition of strychnine or brandy, should be made. Running in the solution by a funnel and tube does very well provided the symptoms of collapse are not urgent and the solution continues to run ; but every now and again, after injecting one or two pints, the funnel and tube fail to act. The cause of this failure apparently lies in some ill-understood condition of the patient's vascular system, as the difficulty persists after the patency of the cannula and tube has been demonstrated and the height of the column of fluid increased to six feet. I have seen this occur on several occasions, on one of which the patient died without the chance which a free supply of saline solution might have given him. In my own practice I always inject it by a cannula fitted to a Higginson's syringe. I have done this frequently, and have never seen a bad result from injecting slowly but forcibly. The objections to this method are, I believe, theoretical. With the Higginson's syringe tied to the handle of a large jug, one pair of hands can easily manage the injection, which is a great advantage when operating in private. After cœliotomy, I have on several occasions filled the peritoneal cavity with hot sterile saline solution, thus providing the patient with an internal hot-water bag, which I find very useful in combating shock.

